# Crisis challenges of small firms in Macao during the COVID-19 pandemic

**DOI:** 10.1186/s11782-020-00094-2

**Published:** 2020-12-14

**Authors:** Jose C. Alves, Tan Cheng Lok, Yubo Luo, Wei Hao

**Affiliations:** City University of Macao, 4th Floor, Luso-Chinese Building, Avenida Padre Tomás Pereira, Macao, China

**Keywords:** Crisis development, Crisis strategies, Small firms, 2019 novel coronavirus disease (COVID-19), Macao

## Abstract

This research develops a framework that combines crisis stages, stakeholder engagement, and crisis challenges. The framework is applied to small firms in Macao during the 2019 novel coronavirus disease (COVID-19) pandemic crisis. We conduct a qualitative study based on semi-structured interviews with the leaders of six small firms in Macao. The findings suggest that the COVID-19 pandemic has turned into a “normal” context, which blurs the traditional crisis termination stage. We also find that participating firms engage more with internal stakeholders than external ones. The strategies adopted by small firms include flexible human resource (HR) practices, cost reduction, enhancing customer relations, and using government support schemes. These strategies are effective in the short term; firms need to pay attention to diversity and learning for the long term.

## Introduction

A crisis refers to “a sudden and unexpected event that threatens to disrupt an organization’s operations and poses both a financial and a reputational threat” (Coombs 2007: p. 163). Such events threaten business goals and lead to prompt response actions (Hermann 1963). Ample evidence exists to suggest that the responses aim to enhance flexibility, learning capabilities, innovation, and customer relations (Herbane 2010, 2013; Hong et al. 2012; Irvine and Anderson 2006) of small firms. There is considerable research on the impact of the crisis on them, particularly regarding risk factors, effects of the crisis, prevention, and response strategies (Bundy et al. 2017; Doern 2016; Doern et al. 2019; Herbane 2010, 2013).

Although there is considerable amount of research on the impact of the crisis on organizations, the research field is fragmented (Bundy et al. 2017). To synthesize the field, Bundy et al. (2017) propose a crisis development model that considers the roles of internal and external stakeholders and how these roles occur at various stages of a crisis. Although this is a useful model to provide a systematic understanding of the crisis, it does not explain how individuals deal with it. Research has particularly suggested that the sense-making perspective adds relevant insights to understanding crisis (Starbuck and Farjoun 2009; Weick 1993, 2010) and studies about crisis leaders (Boin and Hart 2007).

It is worthwhile mentioning several other qualitative studies about the impact of the crisis on small firms, for example, after Hurricane Katrina (Runyan 2006), the 2011 London riots (Doern 2016), and, more recently, entrepreneurial firms in Germany during the COVID-19 pandemic (Kuckertz et al. 2020). However, none of these qualitative studies have adopted an integrative perspective on the crisis, as Bundy et al. (2017) propose. Runyan (2006) and Doern (2016) focus on the internal perspective of firms and only during the crisis. Meanwhile, Kuckertz et al. (2020) gather the internal views of the startups and external organizations (investors, accelerator, association, and corporation) but only during the crisis stage. Thus, there is limited understanding of the dynamics between the internal and external perspectives during a crisis, and how they occur at different crisis stages.

Therefore, the purpose of this study is to adopt an integrated crisis approach to understand how small firms prepare and deal with the COVID-19 pandemic. Our contribution is twofold. First, we establish the connection between the integrative perspective to the crisis (Bundy et al. 2017) and the sense-making approach (Boin and Hart 2007) to propose a new conceptual framework. The integrative perspective captures conditions that shape the crisis (structure), whereas the sense-making approach elucidates how crisis participants work to shape it (agency). Second, we apply the framework to examine small firms in Macao during the COVID-19 pandemic.

This paper is organized as follows. After the introduction, we review the literature to develop a conceptual framework that captures multiple aspects of the crisis. The next section details the methods and procedures used to learn about six small firms in Macao during the COVID-19 pandemic. Subsequently, in the results section, we present the analysis according to the theoretical framework. Finally, we provide discussions, implications and conclusions of the study.

## Theoretical background

### Crisis and small firms

Small business research has recognized the importance of a crisis perspective (Herbane 2010). A recent review of literature on crisis and small- and medium- enterprises (SMEs) finds that most of the publications focus on financial issues (51%), followed by strategy (41%), and institutional environment (8%) (Eggers 2020). According to Eggers (2020), most of the studies that focus on finance are concerned with the consequences of the crisis on small firms, namely the lack of funding and financing sources. The strategy-oriented studies indicate that successful firms adopt a strategy that is both market- and entrepreneurship-oriented during a crisis.

A recent qualitative study about the effect of the COVID-19 pandemic on 16 startups in Germany (Kuckertz et al. 2020) examines how innovative startups deal with the lockdown and the most effective policies. They find that many startups deploy various responses associated with resilience to turn crisis-induced adversity into opportunity. They propose that entrepreneurs who demonstrate flexibility in their business models are likely to access broader emerging opportunities. This finding points to the temporal aspects of the crisis that require further investigation.

Moreover, based on research conducted after the 2004–2012 economic crisis about entrepreneurial culture and the knowledge diversity of small firms in the United Kingdom (Bishop 2019), Kuckertz et al. (2020) argue that adequate entrepreneurial responsiveness cannot be addressed by short-term measures and needs consistent policies. This highlights the importance of considering the temporal perspective of the crisis.

### The crisis development model

Bundy et al. (2017) propose a crisis development model that considers the roles of internal and external stakeholders over three crisis stages: pre-crisis prevention, crisis management, and post-crisis outcomes. Firstly, during the pre-crisis stage, the internal stakeholders understand organizational preparedness, including reliability, culture, structure, and governance mechanisms. At this stage, the organization analyses the external stakeholders’ relationships to identify positive or negative relationships.

The second stage is described as crisis management. The internal perspective addresses managers’ sense-making efforts to resolve the crisis, whereas the external viewpoint evaluates the outsiders’ perception of the crisis. Organizations are usually concerned not only with understanding the nature of the crisis and developing crisis response strategies but also understanding stakeholders’ evaluations of the crisis, their identification with the organization, their power and influence, and crisis spillovers. Finally, in the post-crisis stage, the internal perspective is focused on the organization’s ability to learn. In contrast, the external perspective is concerned with how society will evaluate the organization once the crisis is over.

In summary, the crisis development model proposes a multi-level and -stage framework that provides an integrative perspective of crisis. Given the pervasiveness of the COVID-19 pandemic, we argue that the crisis development model provides insights into the organization–environment dynamics during a crisis. It also highlights the role of leaders but without a sufficiently detailed explanation. To address this limitation, we propose the integration of sense-making concepts with the crisis development model.

### The sense-making approach to crisis

**Fig. 1:**
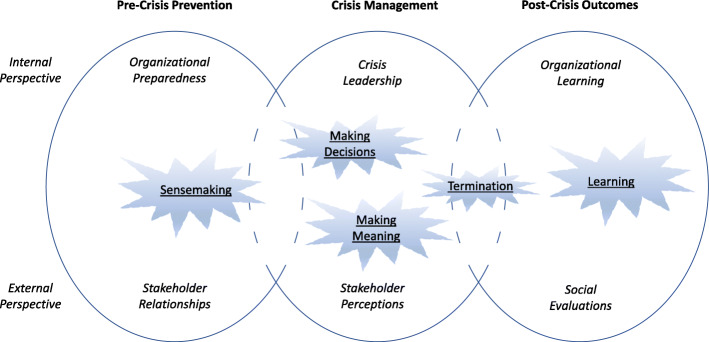
Conceptual model of crisis development and sense-making

Boin and Hart (2007) propose that managing a crisis requires a socially constructed process that addresses five critical challenges: sense-making, decision-making, meaning-making, terminating, and learning. When positioning these five challenges in the crisis development model (Bundy et al. 2017), notably, they occur at different stages of the crisis. Specifically, the sense-making challenge occurs during the pre-crisis stage; the challenges of decision-making, meaning-making, and terminating are faced during the crisis management stage; and the learning challenge in the post-crisis stage. Organizations that develop sense-making are better prepared to overcome crisis challenges (Weick 1993). The integration of crisis development and the sense-making models is represented in Fig. 1.

The remainder of this section describes how sense-making occurs throughout the crisis stages.

#### Sense-making

A crisis begins when a “community of people” perceive an “urgent threat to core values of life-sustaining functions” (Boin and Hart 2007: p. 42) and when they start receiving signals, gathering pieces of information, and appraising events. Maitlis and Christianson (2014: p. 57) also mentioned that a crisis entails evaluating “issues or events that are novel, ambiguous, confusing, or in some other way, violate expectations.” This suggests that leaders have to sense the crisis and gather and evaluate information before forming crisis strategies.

#### Making decisions

Once a community recognizes the existence of a crisis, it addresses the crisis through a combination of cognitive, behavioral, and social processes (Weick 1993). The key feature of crisis decision-making is that it must be an inclusive and collective process. Alternatively, the decisions are likely to be misunderstood and not accepted, making the implementation difficult (Boin and Hart 2007). Leadership effectiveness, teamwork, and decision-making remain critical factors in effective crisis response (Dent et al. 2018).

Unlike rational decision-making, critical decisions are nonlinear (Weick 1993), and instead of being made by small informal groups of leaders, they require diversity and outside crisis specialists (Ruff and Aziz 2003), and networks of people and organizations for effective implementation (Boin and Hart 2007). However, small business research has highlighted that despite the benefits of a systematic approach to planning and decision-making, the individual influence of entrepreneurs is dominant (Herbane 2019).

#### Making meaning

Decision-makers must ensure that people understand and accept decisions without damaging the reputation of those who make them (Coombs 2007). A review of the literature on communication barriers during crisis identifies that crisis obstacles are mostly due to inadequate preparation, particularly the non-acceptance of technology; social barriers due to the diversity of perspectives; and incomplete or poor-quality information (Fischer et al. 2016).

#### Termination

A crisis may be concentrated (e.g., Hurricane Katrina) or extended over a long period (e.g., the COVID-19 pandemic), but will eventually end. Although crisis recovery is never entire and complete (McConnel 2011), crisis leaders need to communicate its closure to “help alleviate continuing anxiety and encourage the return to a state of normality” (Baubion 2013: p. 20). However, if not communicated properly, the closure of a crisis may negatively affect both internal and external stakeholders, including losing key resources and negative reputation; Weick (1995) refers to this as the story that remains.

#### Learning

Research has shown that learning allows firms to cope during the crisis (Weick and Sutcliffe 2011). Battisti et al. (2019) examine the performance of small firms in New Zealand after the 2008 global financial crisis to understand which specific learning mechanism explains the post-crisis resilience of small firms. They find that the owner–manager’s learning goal orientation contributes to long-term sustained performance, whereas practice-based and proximal learning only helps short-term survival. Moreover, they find that knowledge acquisition from proximal and distal sources allows firms with learning orientation to achieve a long-term advantage.

Learning from the crisis is a recursive, emergent, and active process (Elliott and MacPherson 2010). Policymakers and practitioners develop personal coaching and mentoring programs through formal (Battisti et al. 2019) or informal methods (Saunders et al. 2014).

In summary, there is significant research on crisis management that provides insights into the characteristics and evolution of a crisis. However, this research still lacks integration. To address this gap, we construct a crisis framework (Fig. 1) that combines crisis stages and stakeholder engagement (Bundy et al. 2017) with crisis challenges (Boin and Hart 2007). We now apply this framework to explore how small firms in Macao responded to the COVID-19 pandemic.

## Methods

### Research context

The Macao Special Administrative Region (SAR) recorded the first confirmed case on 22 January, 2020. Given Macao’s high urban density and the massive flow of visitors, the government of Macao SAR responded promptly and effectively by adopting a strict quarantine policy to lower the spread of the virus. As of 5 September, 2020, the number of confirmed cases was only 46. The lockdown and social distancing policy brought the entire city to a standstill.

Owing to the adverse impacts of the outbreak on the local economy, the government of Macao SAR announced a series of policies in February and April 2020 to provide economic assistance to residents, including consumer e-vouchers (MOP8,000 or approximately USD1,000 per person), professional tax refund, exemption of property taxes and electricity bills, raising the deduction limit for income tax, temporary exemption of tourism tax, and a one-time cash allowance for qualified employees and small businesses (from MOP50,000 to MOP 200,000 or approximately USD6,250 to USD25,000). Additionally, the government also offered loans for SMEs and provided a guarantee for bank loans for SMEs that qualified.

### Research design

The study of how firms deal with the crisis requires a variety of research designs and methods. Given our interest in why and how managers of small firms experience the crisis and manage it, we decide on a qualitative approach. A limitation of a qualitative study is the time required in representative data collection and analysis. However, a qualitative approach provides the opportunity to gather rich data of unique situations like the COVID-19 pandemic.

It is necessary to access the managers’ and leaders’ perspectives to understand the sense-making process of small firms in the context of the COVID-19 pandemic at different crisis stages. Thus, a qualitative approach using semi-structured interviews is appropriate (Yin 2011).

Definitions of small firms differ widely, most likely due to the size and nature of the respective markets. In this study, we adopt the definition of the Macao Economic Services, which considers SMEs with fewer than 100 employees as eligible for loan applications.

We adopt theoretical and purposive sampling. Firms are selected based on size and impact from the COVID-19 outbreak. We interview the owners or general managers of small firms because of their expert knowledge and understanding of the study (Weiss 1995). We include successful young entrepreneurs and firms that are leaders in their industries to ensure representativeness. Some firms have a long history and prior crisis experience. We select firms using the research team’s contacts and cover the significant industries in Macao, such as hospitality, wholesale and retail, real estate, private education, professional services, and food. We identify the participating firms with alphabets (A-wholesale and retail, B-private education, C-hotel, D-real estate agency, E-law firm, and F-cafeteria) to prevent traceability. Table 1 summarises the information of the six firms.

**Table 1:** Description of participant firms

Business name	Industry	Annual profit (MOP)	Number of employees	Years of history	Licensing and regulatory body
A	Wholesale and retail	Below MOP 1 million	3	2 years	None
B	Private education	Above MOP 1 million	12	38 years	Education and Young Affairs Bureau
C	Hotel	Above MOP 1 million	82	36 years	Macao Government Tourism Office
D	Real estate agency	Above MOP 1 million	7	2 years	Housing Bureau
E	Law firm	Below MOP 1 million	5	5 years	Bar Association
F	Cafeteria	Above MOP 1 million	23	4 years	Municipal Affairs Bureau

### Data collection

We used a semi-structured interview (see the Appendix) divided into three parts: background of the firm, impact of the COVID-19 pandemic on business, and crisis management. We began the interview by asking the participant to describe their firm and followed with an open question: “Can you tell me about your experience during the crisis?” The interviews were conducted as a natural conversation to allow participants to speak openly about the subject. During the interview, we asked probing questions to identify more about the involvement of stakeholders in the crisis and its purpose.

To ensure the study’s validity, we considered recommendations proposed by Yin (1994) and Hong et al. (2012). To address construct-validity, we ran a pilot interview with the owner of another SME on 5 March, 2020. The interviewee considered that the interview questions were clear and without ambiguity. We conducted two rounds of interviews with each participant. The first round was arranged from 8 March, 2020 to 18 April, 2020. We conducted the interviews via social media (WeChat, WhatsApp, and Zoom) to avoid face-to-face contact during the COVID-19. The interviews lasted 60 mins on average and were recorded and stored in digital format in WeChat or WhatsApp voice clips. We also took written notes during the interviews. The second round interview was conducted from 10 May, 2020 to 2 July, 2020, and each lasted approximately 15 mins. All interviews were conducted in Chinese, and the translation into English was done immediately after. To validate the accuracy of the data, we sent summaries of the interviews to the interviewees for confirmation within three days after the interviews. To increase trust with participants, we promised to send them a copy of this research.

We adopted the analytic strategies of pattern-matching and explanation-building to compare data within and across firms (Hong et al. 2012; Yin 1994). The interview data were content analysed. In the first-order analysis, we read the interview transcripts to identify segments of text (categories) representing the experiences of managers associated with the crisis (Table 2). Some segments of data refer to ideas that are not represented in the framework; however, they are also considered in the analysis as new categories. In this step, we also compared the analytical categories across the six firms to identify data patterns (Table 3).

**Table 2:** First-order analysis—analytical categories

Analytical categories	Examples of direct quotations
Planning	“Our existing crisis plan mostly focuses on dealing with HR and customer relations. Crisis planning in other areas are lacking” —A. “We do have a comprehensive set of crisis plans as required by the regulators” —B, C and F. “We do not have any structured crisis management plan as we do not think it is helpful. It would not make a big difference as COVID-19 is a pandemic, and the economic consequence is unavoidable” —D and E.
Testing	“We perform periodic (annually or semi-annually) testing on our existing crisis plan” —B, C and F. “We consider conducting periodic testing is important for our staff to react promptly and appropriately in crises” —C.
Signaling	“Currently, we have a formal crisis signaling mechanism” —A, B, C and F.
Communication among employees	“We have provided a formal crisis management manual to our staff” —B, C, E and F. “We communicate verbally among employees regarding our crisis management strategies” —A. “We update crisis management issues in our monthly staff meetings” —B and C.
Documentation	“We document our crisis management strategies in paper form” —F. “Crisis documents are stored in the electronic form mostly” —A, B, C and E.
Contingency plan	“We do have a contingency plan as required by the regulators” —B, C and F.
Monitoring	“We review our existing crisis management strategies every two years and amend them if needed” —B and C. “We believe that periodic review of crisis management is important as the operation environment keeps changing” —C.
Closedown or shorten operation hours	“In view of the rapid spread of the virus, we decide [d] to close down the shop in January and February” —E and F. “The lockdown is for the benefit of our employees and customers” —A, B and F. “In March, the number of infected has become stable, and we re-open our business but reduce the operation hours” —A, B and F.
Flexible HR policy and employees’ stress management	“Having discussed with our employees, we give them the options of working at home and flexible working hours” —B, C and E. “For employees residing in Chinese mainland, we ask them to stay in Macao or take no paid leave if they decide to stay in Chinese mainland” —C and F. “We appoint a supervisor to be in charge of employees stress management. Employees who have difficulties during the outbreak can consult the supervisor for assistance” —B and C.
Cost cut	“We significantly cut down our labor costs and other operational costs by using flexible HR policies and reducing operation hours” —C and F. “We try to negotiate with our landlord and luckily he reduces our rental by 10% since March” —F.
Reducing inventory	“We stop buying inventories since January as the customer flows have significantly dropped” —A.
Exploring new products	“As the sports stadiums are locked down, the demand for badminton supplies drops dramatically. In view of this, we explore new products such as indoors sports equipment, massage machine, and towels. This helps maintain sales at 40% of the normal sales level and allows us to survive” —A.
Customer relation/advertising/discount promotion	“We give an additional 15% discounts to our customers since March, and the strategy helps restore sales revenue by around 20%” —A and F. “We give a significant discount for long-stay customers, which helps attract companies who need to accommodate their foreign workers. The discount policy is extremely helpful for our survival. Room sales are even higher than that before the outbreak” —C. “We use e-channels such as Facebook and WeChat to advertise and promote our products since February or March, and we successfully draw the attention of our customers. We intend to rely more heavily on this new advertising and promotion channel in the future” —A and D.
Extending credit period with suppliers	“The suppliers are very considerate and give us an additional one-month credit period since February” —A, C and F.
Timely communication among employees	“We hold 30-min live chats twice a week since mid-January for our staff to communicate with the management” —E. “We use Zoom, WeChat and WhatsApp to communicate with our employees on [a] timely basis during the outbreak” —B. “Top management holds online meetings three times a week” —C.
Involvement of stakeholders in the decision-making process	“When making decisions during the outbreak, we consult our staff at different levels. It is a group decision” —B, C and F. “We also consult external stakeholders such as the regulators, customers, and suppliers in the decision process” —C and F.
Restoring public confidence	“Restoring public confidence is most vital for recovery after the outbreak. Macao is famous for its gaming and tourism industries. Whether tourists find it safe to visit the city would have [a] significant impact on the recovery of the economy” —C. “There is nothing much we can do to recover from the crisis. The market relies heavily on the recovery of public confidence” —D.
Industry diversification	“In the long run, we intend to diversify the operating risk by entering new industries such as food and beverage, and arts and entertainment, which are not solely relied on tourist flows” —C. “We are thinking of exploring new market sectors, such as providing professional coaching services for indoor training” —A. “As many of our managers and lecturers are well trained in business and management areas, we have established a consulting firm in May to provide business consulting services for SMEs” —B.
Reforming HR policy	“We decide [d] to reduce our reliance on foreign workers after the outbreak” —F. “After the outbreak, we will focus more heavily on takeaway and food delivery. We may need to hire more part-time helpers and terminate some full-time staff. This can help cut down labor costs by around 20%” —F. “In the future, we would like to hire employees who are well equipped with IT skills” —B and E. “The outbreak has inspires us to adopt flexible working hours and allow employees to work at home as long as they can complete their tasks” —B, E and D. “Our managers will keep closer communication with our staff using social platforms” —B, C and E.
Investing in learning on crisis management	“We intend to invest in learning on crisis management after the outbreak, and hopefully, this may help reduce our loss in the next crisis” —B, C, E and F. “We do not have an exact budget for the learning through” —E. “We intend to invest around MOP10,000 (USD1,250) annually on strengthening crisis management” —B. “Estimated budget of learning on crisis management is around MOP20,000 (USD2,500) annually” —F. “Our hotel plans to increase investment in training for crisis management and stress management, with an estimated annual investment amounting to MOP200,000 (USD 25,000)” —C.
Investing in learning technology	“During the outbreak, we conduct our management meetings through Zoom, WeChat, and WhatsApp, and we find it highly efficient. We decide to go on with this even after the outbreak” —B, C and E. “We switch our operation to online sales during the outbreak and find it helpful to maintain sales revenue. In the future, we will focus more on online sales mode. Our staff need to be trained in e-commerce and technology to cope with the change in our business model” —A and D. “The COVID-19 outbreak has significantly changed the business model, and we intend to invest in technology, such as to film our products and conduct more online live chats” —A and D. “Because of the outbreak, we deliver some of our courses online through Zoom. This inspires us to develop our online course department in the long run so that students who live far away can easily join our online courses at their convenience” —B.
Investing in health and safety equipment	“We renovate our safety equipment (fire alarm, etc.) during the outbreak when there is no customer” —B and C.
Redecoration	“We redecorate our C in the early stage of the outbreak as there are fewer customers and the redecoration would not be too disturbing to them. We believe the renovation can enhance our competitiveness after the outbreak” —C.
Applying for SMEs loan from the government	“We have applied for the special loan for SMEs from the Macao Economic Services. We intend to repay the loan within two or three years” —A and D. “The SMEs loan may not be too helpful as it creates additional financial stress in the coming years” —C.

**Table 3:** First order analysis—Identification of patterns

Stage / Categories	Internal vs. External	Firm A	Firm B	Firm C	Firm D	Firm E	Firm F	Total
***Pre-crisis***								
Planning	Internal	√	√	√			√	4
Testing	Internal		√	√			√	3
Signaling	Internal	√	√	√			√	4
Communication with employees	Internal	√	√	√		√	√	5
Documentation	Internal	√	√	√		√	√	5
Contingency plan	Internal	√	√	√		√	√	5
Monitoring	Internal		√	√		√	√	4
***During crisis***								
Shorten operation hours	Internal	√	√	√	√	√	√	6
Flexible HR policy and employees’ stress management	Internal	√	√	√	√	√	√	6
Cost reduction	Internal	√	√	√	√	√	√	6
Reducing inventory	Internal	√		√			√	3
Exploring new products	Internal and External	√		√			√	3
Customer relation, advertising, discount promotion	External	√	√	√	√	√	√	6
Extending credit period with suppliers	External	√		√			√	3
Timely communication with employees	Internal		√	√	√	√		4
Investing in health and safety	Internal		√	√				2
Redecoration	External		√	√				2
Government loans	External	√			√			2
Investing in learning on crisis management	Internal	√	√	√		√	√	5
Investing in learning technology	Internal	√	√	√	√	√	√	6
Restoring public confidence	External	√	√	√	√	√	√	6
Diversification	Internal and external	√	√	√			√	4

## Results

The empirical results of the study are presented according to the three stages of the crisis, and we describe the various crisis challenges within each stage.

### Stage 1: pre-crisis prevention

#### Challenge of sense-making

The challenge of sense-making refers to threats to life-sustaining functions (Boin and Hart 2007). We find major challenges in this category, including economic losses, perceived duration of the crisis, and preparedness.

All participants suffered temporary closedown of the business and reported economic losses (ranging from MOP100,000 to MOP500,000, or from USD12,500 to USD62,500) during the crisis. The most significant economic impact was the loss of customer flow (demand constraint) instead of cash flow difficulty (financial constraint). The firms expect that adverse effects will last far beyond the period of the actual COVID-19 pandemic. Initially, they estimated a recovery time ranging from 3 to 36 months, but in the second interview, the estimates increased to 18–48 months. The long timespan of the COVID-19 crisis makes the respondents think that strategies that worked in previous crises may not work this time.

*“The COVID-19 does not resemble any other crises that we have encountered before. We cannot see an end to it. The pandemic may be around for one or two years or even much longer”* — *C.*

Leaders of firms B, C, and F are more prepared for the crisis. These firms have a long history, a larger number of employees, and more government regulations. The leaders, having experienced previous crises, use the experience to develop a crisis management strategy. Compared with previous crises such as flooding and customers’ complaints, the leaders of the participating firms failed to detect the early signals of the epidemic until mid-January 2020.

*“The COVID-19 comes up quite unexpectedly, and there is no early warning signal. Our operation is seriously disrupted”*—*B.*

Without a formal crisis plan, the SME firms have a weaker market position, fewer resources, and lacking a sense of crisis.

*“We do not have any structured crisis management plan as we do not think it is helpful. It would not make a big difference as COVID-19 is a pandemic, and the economic consequence is unavoidable”* —*D and E.*

Firms B, C, and F operate in highly regulated industries and must have a structured crisis management plan to file with the regulators. Periodic testing of the plan involving various stakeholders such as employees and customers is also required.

*“We consider conducting periodic testing is important for our staff to react promptly and appropriately in crises”*—*C.*

However, the potential of a sound crisis management system on preventing events such as COVID-19 or reducing potential losses is uncertain. All respondents believe that even structured crisis planning and the ability to sense the crisis at an early stage would not have helped much in this crisis, unlike in other classes of crises. However, previous research has found that small firms with better preparedness and sense-making may recover sooner from stress (Irvine and Anderson 2006).

### Stage 2: crisis management

#### Challenge of making decisions

Once managers identify the crisis, they also start making decisions. Our data suggest that SME managers seek opinions from other persons and institutions before making decisions and have emphasized short-term strategies. These strategies tend to focus on achieving economic sustainability through increased engagement and planning procedures, slimming operations, and the introduction of flexible HR practices, diversification, and new digital strategies.

Most of the firms interviewed involve both internal and external stakeholders in the crisis decision process. Of the six firms, firm D is the only one that does not involve external stakeholders. Nevertheless, none of them indicate involving an outside crisis expert in the existing crisis mechanism, as in Ruff and Aziz (2003).

*“When making decisions during the outbreak, we consult our staff at different levels. It is a group decision”* —*B, C, and F.*

*“We also consult external stakeholders such as the regulators, customers, and suppliers in the decision process”* —*C and F.*

All participating firms, except firm D, emphasize crisis planning procedures. From the follow-up interviews, we find that these firms show significant signs of recovery 4 months after the outbreak.

*“We have provided a formal crisis management manual to our staff”* — *B, C, E, and F.*

*“We update crisis management issues in our monthly staff meetings”*—*B and C.*

The leaders of the six firms quickly re-organize operation procedures, namely changing operating hours and reducing costs.

*“In March, the number of infected has become stable, and we re-open our business but reduce the operating hours”*—*A, B, and F.*

*“We stop buying inventories since January as the customer flows have significantly dropped”*—*A.*

*“We significantly cut down our labor costs and other operational costs by using flexible HR policies and reducing operating hours”*—*C and F.*

Firms recently set up, especially those founded by young entrepreneurs and in small size, show more flexibility and prompt reactions during the COVID-19 outbreak. Five small firms founded by young entrepreneurs quickly change their HR policies.

*“Having discussed with our employees, we give them the options of working at home and flexible working hours”*—*B, C and E.*

The pandemic also inspire the participating firms to reform their HR policies in the long run. The leaders foresee that the market will be different, and what worked in the past may not work in the future. The firms cut down their reliance on foreign workers and recruit new local workers with information and technology (IT) skills.

*“After the outbreak, we will focus more heavily on takeaway and food delivery. We may need to hire more part-time helpers and terminate some full-time staff. This can help cut down labor costs by around 20%”*—*F.*

*“In the future, we would like to hire employees who are well equipped with IT skills”* —*B and E.*

We find the emphasis on product- and industry- diversifications (four out of six) regarding marketing strategies. Some firms, such as A, C, and F, intend to diversify into completely different industries that do not rely on tourist flow. The leaders are concerned that the pandemic may last longer than expected, significantly reducing demand in the tourism-related sector. This trend has not been detailed in the prior literature.

*“In the long run, we intend to diversify the operating risk by entering new industries such as food and beverage, and arts and entertainment, which are not solely relied on tourist flows”* —*C.*

*“We are thinking of exploring new market sectors, such as providing professional coaching services for indoor training”* —*A.*

*“As many of our managers and lecturers are well trained in business and management areas, we have established a consulting firm in May to provide business consulting services for SMEs”* —*B.*

Three of the firms have also reduced inventory and explored new products.

*“As the sports stadiums are locked down, the demand for badminton supplies drops dramatically. In view of this, we explore new products such as indoor sports equipment, massage machines, and towels. This helps maintain sales at 40% of the normal sales level and allows us to survive”*—*A.*

All the interviewed firms have enhanced their networking with customers by increasing advertising with technology (using e-channels such as Facebook and WeChat) and offering discounts during the crisis.

*“We use e-channels such as Facebook and WeChat to advertise and promote our products since February or March, and we successfully draw the attention of our customers. We intend to rely more heavily on this new advertising and promotion channel in the future”* —*A and D.*

A few firms have also reduced prices during the outbreak as a way to attract customers.

*“We give an additional 15% discounts to our customers since March, and the strategy helps restore sales revenue by 20%”*—*A and F.*

*“We give a significant discount for long-stay customers, which helps attract companies who need to accommodate their foreign workers. The discount policy is extremely helpful for our survival. Room sales are higher than that before the outbreak”* —*C.*

These strategies are also likely to evolve in the future. Some firms are interested in changing their business model to expand into e-commerce (firms A, B, and D).

*“Because of the outbreak, we deliver some of our courses online through Zoom. This inspires us to develop our online course department in the long run so that students who live far away can easily join our online courses at their convenience”* —*B.*

Of the six firms, two intend to invest in hardware (health and safety equipment and redecoration). Two firms are interested in using SME loans from the government. Three of them (firms A, C, and D) indicate that loan repayments would exert financial pressure on the organizations.

*“We have applied for the special loan for SMEs from the Macao Economic Services. We intend to repay the loan within two or three years”* —*A and D.*

*“The SMEs loan may not be too helpful as it creates additional financial stress in the coming years”* —*C.*

#### Challenge of making meaning

During a crisis, small firms need to make decisions and ensure that people understand and accept those decisions without damaging their reputation. Therefore, timely and effective communication is paramount. Except for firms A and F, the leaders have maintained timely communication with their employees throughout the crisis. Firms have used social media such as WeChat, WhatsApp, and Zoom, to hold staff meetings. The leaders find these communication channels effective and efficient and indicate that they will continue to use them in the future.

*“We hold 30-minutes live chats twice a week since mid-January for our staff to communicate with the management”* —*E.*

*“During the outbreak, we conduct our management meetings through Zoom, WeChat, and WhatsApp, and we find it highly efficient. We decide to go on with this even after the outbreak”* —*B, C and E.*

Besides communicating with their employees, most participating firms have maintained frequent communications with external stakeholders, namely customers, to disseminate company policy promptly. Firms B, C, and F also work closely with the regulatory agencies. Firms A, C, and F negotiate with their credit suppliers for extended credit periods.

*“The suppliers are very considerate and give us an additional one-month credit period since February”*—*A, C and F.*

#### Challenge of terminating

After almost 9 months, the COVID-19 pandemic has yet to end. However, leaders and the public have started to interpret the long-term crisis as becoming normal. Although this “normal” situation is still evolving, it also signals the intention to return to normalcy. All participating firms believe that the most vital task in the aftermath is to restore public confidence. This can be achieved by the cooperation of the government and society at large.

*“Restoring public confidence is most vital for recovery after the outbreak. Macao is famous for its gaming and tourism industries. Whether tourists find it safe to visit the city would have [a] significant impact on the recovery of the economy”* —*C.*

*“There is nothing much we can do to recover from the crisis. The market relies heavily on the recovery of public confidence”* —*D.*

As the pandemic is enduring longer than expected, the leaders have found that they must make decisions over a relatively long span. The leaders believe that it is more important to act swiftly and gradually cope with the development of the pandemic. As the impact of the crisis is the continued low demand, firms have adopted strategies to achieve resilience, such as unusual non-paid leave for their employees.

*“For employees residing in Chinese mainland, we ask them to stay in Macao or take no paid leave if they decide to stay in Chinese mainland”*—*C and F.*

#### Challenge of organizational learning

The managers’ learning goal orientation and knowledge acquisition result in long-term advantage, whereas practice-based and proximal learning have a short-term effect on performance (Battisti et al. 2019).

All interviewed firms express the intention to invest in technology. The leaders foresee that technology will contribute significantly to HR management and marketing in the post-crisis era. Except for firm F, all the firms intend to explore new market opportunities with technology. However, small firms are more hesitant to make large investments in learning.

Five participant firms are willing to invest in learning crisis management. Firms B and C, which have adopted learning in their survival strategies, seem to have recovered faster than others.

*“Our hotel plans to increase investment in training for crisis management and stress management, with an estimated annual investment amounting to MOP 200,000 (USD25,000)”* —*C.*

*“We switch our operation to online sales during the outbreak and find it helpful to maintain sales revenue. In the future, we will focus more on online sales mode. Our staff need to be trained in e-commerce and technology to cope with the change in our business model”* —*A and D.*

*“The COVID-19 outbreak has significantly changed the business model, and we intend to invest in technology, such as to film our products and conduct more online live chats”* —*A and D.*

## Discussion

In this section, we provide an in-depth discussion of the results. Table 4 shows our interpretation of the findings according to crisis stages, challenges, and stakeholders.

**Table 4:** Crisis challenges and strategies

Crisis challenges	Crisis strategies (number of firms)
***Pre-crisis***	
Sense-making	Planning (4) Testing (3) Signaling (4) Documentation (5) Contingency plan (5) Monitoring (4) Communication with employees (5)
***During crisis***	
Making decisions	Cost reduction (6) Closedown or shorten operation hours (6) Flexible HR policy and employees’ stress management (6) Reducing inventory (3) Exploring new products (3) Customer relations, advertising, discount promotion (6) Extend credit period with suppliers (3)
Making meaning	Timely communication with employees (4) Reform HR policy (2) Diversification (4)
Termination or become normal	Restore public confidence (6) Extended non-paid leave (2) Invest in health and safety equipment (2) Redecoration (2) Apply for SMEs loan from the government (2)
Organizational learning	Strengthen crisis management strategies (4) Investing in learning on crisis management (5) Investing in learning on technology (6)

### Sense-making

Among the six participants, three have made extensive preparations for a crisis (private education, hotel, and cafeteria), two have moderate preparations (wholesale and retail, and law firm), and one has made no preparation (real estate agency) (see Table 3). This suggests that businesses that require higher customer engagement and are more regulated are likely to make more detailed preparations for crisis and are alert. This raises the question of whether further crisis prevention regulations should be developed for these sectors.

### Making decisions

Most of the firms implement a series of decisions to reduce operational costs, in sectors such as inventory and labor, on recognizing the crisis. They also extend credit to suppliers, introduce new products and services, and increase promotions to attract local customers. All these are quick and short-term actions that, despite having immediate benefits, may not be sufficient for the long term.

### Making meaning

Except for the wholesale and retail and cafeteria firms, all others have maintained timely communication with employees during the crisis, to share information about the operational decisions. All six firms are active in their communication with customers. However, only three have provided extended credit to suppliers (wholesale and retail, hotel, and cafeteria), and two are considering the use of government loans (wholesale and retail, and real estate agency). This indicates that service firms are less likely to extend credit and that interest in government loans is concentrated in certain sectors only.

### Termination as normal

Evidence from Hong Kong SAR and other jurisdictions indicates the possibility of a second and third wave of the crisis and more. Therefore, instead of the traditional crisis development model of dissecting the crisis stages into pre-crisis, during-crisis, and post-crisis, the results indicate that the borderline between the during-crisis and the post-crisis stages has blurred. After the first outbreak, people think that the crisis is over and start to adopt post-crisis strategies, but they soon observe the second wave and return to the during-crisis stage. The normal feature of COVID-19 requires new insights on how to dissect crisis into stages.

### Learning

All the interviewed firms express interest in investing in learning on crisis management and technology. Three firms mention their plans to change their business model to incorporate e-commerce. This requires employees to acquire new skills and the firms’ willingness to invest in training. Two firms (law firm and real estate agency) do not have a budget for learning programs and are considering investing in short-term learning. However, certain firms express concerns regarding this kind of training.

### Stakeholders

Most of the participating firms involve internal and external stakeholders in their decision process, with more reliance on internal stakeholders. One of the firms (real estate agency) relies only on internal stakeholders. External stakeholders are limited to customers, regulators, and suppliers. Other external stakeholders are involved only to a small extent. For example, none of the firms mention seeking help from outside crisis specialists.

### Diversification

Our results indicate that some firms (wholesale and retail, private education, and hotel) have mentioned industry diversification as a survival and resilience strategy. The industry diversification strategy aims to reduce the firms’ reliance on the flow of tourists to diversify business risk. The hotel intends to explore new market sectors such as food and beverage, arts and entertainment—the wholesale and retail business plans to provide professional coaching services for indoor sports. The educational institution has set up a consulting firm providing business advisory services to local SMEs. In their industry diversification plans, the firms often mention the use of technology, such as social media like Facebook and WeChat, to promote their products or services. This finding has not been mentioned in previous research.

## Implications

### Theoretical implications

Our study has various implications for the management literature on the crisis. First, we propose a framework that integrates the crisis development model (Bundy et al. 2017) and the sense-making approach (Boin and Hart 2007). This model responds to crises researchers’ calling for studies that capture the complexity, processes, and experiences.

Our findings also show that the traditional crisis development model proposed by Bundy et al. (2017) does not explain the termination stage of the COVID-19 pandemic. In the current COVID-19 crisis, the termination stage has been reinterpreted by the public and participant firms as becoming normal, which the end cannot be seen. Evidence from several regions and countries suggests a third wave and more rounds to come. The uniqueness of COVID-19 has blurred the borderline between the during- and post-crisis stages.

### Practical implications

Small firms in Macao tend to involve internal and external stakeholders in the crisis decision process, except for the real estate agency, which mainly relies on internal stakeholders. However, even for firms with external stakeholders, this engagement is limited to customers, regulators, and suppliers. This indicates the need for procedures and regulations that encourage broader communication processes.

Small firms may be flexible (Doern et al. 2019; Simón-Moya et al. 2016) and with low bureaucracy (Hong et al. 2012; Irvine and Anderson 2004, 2006; Spillan and Ziemnowicz 2003). However, they lack formal crisis management processes, especially in new firms with little previous crisis experience. Small businesses may consider long-term strategies focusing on financial and non-financial factors.

Corporate social responsibility (CSR) strategies and compliance depend on firm size (Baumann-Pauly et al. 2013; Ketola et al. 2009). CSR is a double-edged sword. It helps promote the firm’s image but may set boundaries that prohibit large firms from swiftly and effectively restructuring their policies, such as reducing labor during the crisis. This occurred in the gaming and hospitality industry when the Macao SAR government persuaded large firms not to terminate employment in the early stage of the outbreak. The issue of the balance among feasibility, responsibility, and sustainability remains.

Among the participating firms, we did not find any firm that has involved external crisis specialists in the management process. This limitation suggests a potential disengagement with external stakeholders and the need to develop a broader crisis management plan (Dent et al. 2018). Small firms should work closely with various stakeholders to recover and survive the crisis (Brunet and Houbaert 2007).

All firms are interested in exploring new markets and incorporating technology into their future growth. This is a sign of active learning and improvement. In the long run, a strong driving force for small firms to learn and innovate may be their urge to grow and expand their market (Bullough and Renko 2013; Elliott 2009; Saunders et al. 2014).

Leaders of the participating firms do not believe that the sense-making challenge (crisis identification) will help as the COVID-19 pandemic is unique, and do not find the experience of previous crises helpful. They consider that decision-making and learning are vital during the current crisis. They emphasize certain crisis challenges and neglect others as a warning for future crisis preparation.

All firms are satisfied with the existing government policies. They believe that the proposed initiatives are helpful. However, small firms must understand that these short-term policies have limitations, and there is a need for continuous renewal and learning (Battisti et al. 2019; Bishop 2019). The leaders foresee that technology will change internal communication and marketing during and after the crisis. It is, therefore, crucial that small firms cope with technological advancement.

As previous research has shown, the tourism industry is highly vulnerable to public health crises (Irvine and Anderson 2004, 2006; Jonas et al. 2011; Wilks et al. 2006). Thus, with an economy heavily focuses on tourism and peripheral industries, Macao needs to increase diversity. Small firms need to develop their capacity for pivoting and adapting their business models.

### Policy implications

The participant firms operate with short-term orientation. They show flexibility, effectiveness in adopting solutions, and assume that the positive pre-crisis environment is likely to resurge in time. Moreover, they hesitate about investment. Most of them intend to invest in crisis management and technology training, but only a few of them plan to use government loans.

The combination of short-term orientation and investment hesitation is likely to have a long term impact on small firms. Battisti et al. (2019) find that the survival and growth of small firms after a crisis is associated with managers’ learning orientation. They recommend policies centered on the person rather than on the firm, specifically for programs promoting cognitive and behavioral learning; small firms in Macao will benefit from similar policies. This training helps firms increase the sources and types of new knowledge, which may help managers consider new business diversification.

Another type of policy refers to the regulatory and licensing requirements of small service firms. Although small service firms operate with fewer policy restrictions, firms in these sectors are more prepared to deal with a crisis. Thus, firms’ preparedness suggests that expanding regulatory requirements to more service sectors may also help deal with a crisis. Lastly, there is a need for policies and mechanisms that put the small firms in contact with a broader number and range of stakeholders, including specialists.

### Limitations

Due to the lack of a uniform and standardized definition of SMEs across economies, care must be taken when applying our findings to SMEs in other regions. Their size and resources may not be comparable. This study focus on Macao, an economy with ample financial resources due to high gaming and tourism tax income. The reaction of small firms operating in other contexts should be studied further. Moreover, although we carefully select our participating firms to enhance our sample representativeness, the small sample size may have limited generalizability.

## Conclusion

This study proposes a conceptual framework to explain crisis development stages and crisis challenges. We provide insights into how small businesses in Macao have interpreted and responded to the COVID-19 pandemic. Due to the continual pandemic crisis, the borderline between the during- and post-crisis stages has blurred. The challenge of crisis termination has been reinterpreted as a “normal” stage (Välikangas and Lewin 2020), a new concept that deserves further research and discussion. However, some of the strategies followed in previous crises may still be useful for small firms; the uniqueness of the COVID-19 pandemic requires them to expand stakeholder engagement and develop new learning.

## Data Availability

Summary of the data collected is presented in the tables. Details of data and materials are available upon request. Please contact Tan Cheng Lok at stellalok@chwcpa.com.mo.

## Appendix

### Semi-structured interview questionnaire

Interviewer: Date: Firm:

### Opening remarks

Greetings. Thanks for participating in this survey. Stating the purpose of this survey.

### Part 1: Demographic information

Q1 Name of the organization:

Q2 Name of the interviewee:

Q3 Position of the interviewee:

Q4 History of the firm:

Q5 Business form:

Q6 Industry:

Q7 Main products:

Q8 Number of employees:

Q9 Is there any non-Macao resident under your current employment?

Q10 Annual revenue in MOP:

Q11 Main customers (cash or credit basis):

Q12 Main suppliers (cash or credit basis):

### Part 2: Economic impacts of the COVID-19 outbreak

Q1 How is the operation of your organization impacted by the COVID-19 outbreak?

Q2 What is the most urgent difficulty faced by your organization currently?

Q3 What is the estimated financial loss of your organization in this COVID-19 crisis?

Q4 Do you think firm size would have any impact on the economic loss in this COVID-19 crisis? (This question was asked in the follow-up interviews on May 10–July 2, 2020).

Q5 As the economy returns to normal, how has the firm performed in April and May 2020 as a comparison to February and March 2020 when the crisis was at its peak?

(This question was asked in the follow-up interviews on May 10–July 2, 2020).

### Part 3: Crisis management strategies

#### The experience

Q1 During the working period, have you ever experienced any crisis? What types of crises have you experienced?

Q2 In your opinion, how does COVID-19 differ from other crises that you have encountered so far? Do you think the crisis management strategies that work in previous crises also work in this crisis? (This question was asked in the follow-up interviews on May 10–July 2, 2020).

#### Preparation

Q3 Does your organization have a crisis management plan and strategy?

Q4 Have you been involved in any crisis management mechanisms?

Q5 Is an outside crisis specialist involved in the crisis management mechanisms?

Q6 How does your organization identify any potential crisis before it occurs?

Q7 Does your organization have a contingency plan to prevent the crisis or minimize the negative impacts of the crisis?

Q8 Do you have a formal documentation mechanism in the COVID-19 crisis?

#### Survival strategy

Q9 How does your organization respond to the current COVID-19 outbreak?

Q10 Did you work with your customers, suppliers, employees, landlord, and other internal or external stakeholders to deal with this crisis? What is the effect?

Q11 Do you think those actions are useful for survival in this crisis event?

Q12 In your opinion, what else could have been done?

Q13 Do you think firm size would make any difference in the survival strategy in your industry? (This question was asked in the follow-up interviews on May 10–July 2, 2020).

#### Recover strategy

Q14 How long do you believe it will take to recover from this crisis fully?

Q15 After the COVID-19 crisis, what does your organization plan to do to recover from it?

Q16 What are the major barriers or difficulties for recovery? How are you going to overcome these?

Q17 Once the COVID-19 outbreak is over, does your organization plan to make any modification in your products and services, operation process, or market position to prevent similar crises in the future?

Q18 After the COVID-19 crisis, is your organization willing to invest in learning and training in crisis management? To what extent?

Q19 What is a future outlook about the possible crisis events for your organization?

Q20 Do you find your recovery strategies useful from the performance of your firm in April and May? (This question was asked in the follow-up interviews on May 10–July 2, 2020).

Q21 Do you think firm size would make any difference in the recovery strategies in your industry? (This question was asked in the follow-up interviews on May 10–July 2, 2020).

Q22 In the decision-making process, have you discussed with your employees and or other stakeholders? (This question was asked in the follow-up interviews on May 10–July 2, 2020).

#### Policy

Q23 Is your organization satisfied with the existing government policies for the COVID-19 outbreak?

Q24 In your opinion, how can the government of Macao SAR help your organization recover from the COVID-19 crisis?

End of interview.

Your participation in this survey is highly appreciated.

## Abbreviations

SME: Small- and medium-sized enterprises
Macao SAR: Macao Special Administrative Region

## References

[CR1] Battisti M, Beynon M, Pickernell D, Deakins D (2019). Surviving or thriving: The role of learning for the resilient performance of small firms. Journal of Business Research.

[CR2] Baubion, C. (2013). *OECD risk management: strategic crisis management*, *OECD working papers on public governance, no. 23*. OECD Publishing. 10.1787/5k41rbd1lzr7-en.

[CR3] Baumann-Pauly, D., Wickert, C., Spence, L. J., & Scherer, A. G. (2013). Organizing corporate social responsibility in small and large firms: Size matters. *Journal of Business Ethics*, *115*(4), 693–705.

[CR4] Bishop, P. (2019). Knowledge diversity and entrepreneurship following an economic crisis: An empirical study of regional resilience in Great Britain. *Entrepreneurship and Regional Development*, *31*(5–6), 496–515.

[CR5] Boin A, Hart PT, Rodriguez H, Quarentelli EL, Dynes RR (2007). The crisis approach. Handbook of disaster research.

[CR6] Brunet S, Houbaert P (2007). Involving stakeholders: The Belgian fowl pest crisis. Journal of Risk Research.

[CR7] Bullough A, Renko M (2013). Entrepreneurial resilience during challenging times. Business Horizons.

[CR8] Bundy J, Pfarrer MD, Short CE, Coombs WT (2017). Crises and crisis management: Integration, interpretation, and research development. Journal of Management.

[CR9] Coombs WT (2007). Protecting organization reputations during a crisis: The development and application of situational crisis communication theory. Corporate Reputation Review.

[CR10] Dent, P., Woo, R., & Cudworth, P. (2018). *Crisis management for the resilient enterprise*. Deloitte Insights https://www2.deloitte.com/ch/en/pages/risk/articles/crisis-management-for-the-resilient-enterprise.html.

[CR11] Doern, R. (2016). Entrepreneurship and crisis management: The experiences of small businesses during the London 2011 riots. *International Small Business Journal*, *34*(3), 276–302.

[CR12] Doern, R., Williams, N., & Vorley, T. (2019). Special issue on entrepreneurship and crises: Business as usual? An introduction and review of the literature. *Entrepreneurship and Regional Development*, *31*(5–6), 400–412.

[CR13] Eggers F (2020). Masters of disasters? Challenges and opportunities for SMEs in times of crisis. Journal of Business Research.

[CR14] Elliott, D. (2009). The failure of organizational learning from crisis—A matter of life and death? *Journal of Contingencies & Crisis Management*, *17*(3), 157–168.

[CR15] Elliott, D., & MacPherson, A. (2010). Policy and practice: Recursive learning from crisis. *Group and Organization Management*, *35*(5), 572–605.

[CR16] Fischer, D., Posegga, O., & Fischbach, K. (2016). *Communication barriers in crisis management: A literature review*. Research papers. 168. http://aisel.aisnet.org/ecis2016_rp/168.

[CR17] Herbane B (2010). Small business research: time for a crisis-based view. International Small Business Journal.

[CR18] Herbane B (2013). Exploring crisis management in UK small-and-medium-sized enterprises. Journal of Contingencies & Crisis Management.

[CR19] Herbane B (2019). Rethinking organizational resilience and strategic renewal in SMEs. Entrepreneurship and Regional Development.

[CR20] Hermann CF (1963). Some consequences of crisis which limit the viability of organisations. Administrative Science Quarterly.

[CR21] Hong, P. C., Huang, C., & Li, B. (2012). Crisis management for SMEs: insights from a multiple-case study. *International Journal of Business Excellence*, *5*(5), 535–553.

[CR22] Irvine, W., & Anderson, A. R. (2004). Small tourist firms in rural areas: Agility, vulnerability, and survival in the face of crisis. *International Journal of Entrepreneurial Behavior and Research*, *10*, 229–246.

[CR23] Irvine, W., & Anderson, A. R. (2006). The impacts of foot and mouth disease on a peripheral tourism area: The role and effect of crisis management. *Journal of Travel & Tourism Marketing*, *19*(2–3), 47–60.

[CR24] Jonas, A., Mansfeld, Y., Paz, S., & Potesman, I. (2011). Determinants of health risk perception and how they shape tourists’ potential risk-taking behavior: A case study of low-risk-taking tourists. *Journal of Travel Research*, *50*(1), 87–99.

[CR25] Ketola, T., Blombäck, A., & Wigren, C. (2009). Challenging the importance of size as determinant for CSR activities. *Management of Environmental Quality*, *20*(3), 255–270.

[CR26] Kuckertz, A., Brändle, L., Gaudig, A., Hinderer, S., Reyes, C. A. M., Prochotta, A., & Berger, E. S. (2020). Startups in times of crisis—A rapid response to the COVID-19 pandemic. *Journal of Business Venturing Insights*, *13*, e00169.

[CR27] Maitlis, S., & Christianson, M. (2014). Sense-making in organizations: Taking stock and moving forward. *Academy of Management Annals*, *8*(1), 57–125.

[CR28] McConnel J (2011). Remembering the 1605 gunpowder plot in Ireland, 1605–1920. Journal of British Studies.

[CR29] Ruff P, Aziz K (2003). Managing communications in a crisis.

[CR30] Runyan, R. C. (2006). Small business in the face of crisis: Identifying barriers to recovery from a natural disaster. *Journal of Contingencies & Crisis Management*, *14*(1), 12–26.

[CR31] Saunders, M. N., Gray, D. E., & Goregaokar, H. (2014). SME innovation and learning: The role of networks and crisis events. *European Journal of Training and Development*, *38*(1–2), 136–149.

[CR32] Simón-Moya, V., Revuelto-Taboada, L., & Ribeiro-Soriano, D. (2016). Influence of economic crisis on new SME survival: Reality or fiction? *Entrepreneurship & Regional Development*, *28*(1–2), 157–176.

[CR33] Spillan JE, Ziemnowicz C (2003). Strategic management in small retail businesses. International Small Business Journal.

[CR34] Starbuck, W., & Farjoun, M. (Eds.) (2009). *Organization at the limit: Lessons from the Columbia disaster*. Hoboken: Wiley.

[CR35] Välikangas L, Lewin AY (2020). The lingering new normal. Management and Organization Review.

[CR36] Weick KE (1993). The collapse of sense-making in organizations: The Mann Gulch disaster. Administrative Science Quarterly.

[CR37] Weick KE (1995). Sense-making in organizations.

[CR38] Weick KE (2010). Reflections on enacted sense-making in the Bhopal disaster. Journal of Management Studies.

[CR39] Weick, K. E., & Sutcliffe, K. (2011). *Managing the unexpected: Resilient performance in an age of uncertainty*, (vol. 8). Hoboken: Wiley.

[CR40] Weiss, R. S. (1995). *Learning from strangers. The art and method of qualitative interview studies*. New York: Free Press.

[CR41] Wilks, J., Pendergast, D., & Leeggat, P. (2006). *Tourism in turbulent times: Towards safe experiences for visitors*. Oxford: Elsevier.

[CR42] Yin, R. K. (1994). *Case study research: Design and methods*. Thousand Oaks: Sage Publication.

[CR43] Yin RK (2011). Qualitative research from start to finish.

